# A large national outbreak of COVID-19 linked to air travel, Ireland, summer 2020

**DOI:** 10.2807/1560-7917.ES.2020.25.42.2001624

**Published:** 2020-10-22

**Authors:** Nicola Murphy, Máirín Boland, Niamh Bambury, Margaret Fitzgerald, Liz Comerford, Niamh Dever, Margaret B O’Sullivan, Naomi Petty-Saphon, Regina Kiernan, Mette Jensen, Lois O’Connor

**Affiliations:** 1 Department of Public Health, HSE-South, Cork, Ireland; 2 These authors contributed equally; 3 Department of Public Health, HSE-East, Dublin, Ireland; 4 Health Protection Surveillance Centre (HPSC), Dublin, Ireland; 5 Department of Public Health, HSE-Northwest, Sligo, Ireland; 6 Department of Public Health, HSE- West, Galway, Ireland

**Keywords:** COVID-19, SARS-CoV-2, in-flight transmission, aircraft, passenger, flight

## Abstract

An outbreak of 59 cases of coronavirus disease (COVID-19) originated with 13 cases linked by a 7 h, 17% occupancy flight into Ireland, summer 2020. The flight-associated attack rate was 9.8–17.8%. Spread to 46 non-flight cases occurred country-wide. Asymptomatic/pre-symptomatic transmission in-flight from a point source is implicated by 99% homology across the virus genome in five cases travelling from three different continents. Restriction of movement on arrival and robust contact tracing can limit propagation post-flight.

Air travel has accelerated the global pandemic, contributing to the spread of coronavirus disease (COVID-19) throughout the world. We describe an outbreak that demonstrates in-flight transmission, providing further evidence to add to the small number of published studies in this area. This study depicts the nature of transmission on board, despite implementation of non-pharmaceutical interventions. We demonstrate widespread in-country transmission as a result of imported infection and give recommendations to reduce the risk of importation, and to curtail onwards spread.

## Outbreak description

**Figure 1:**
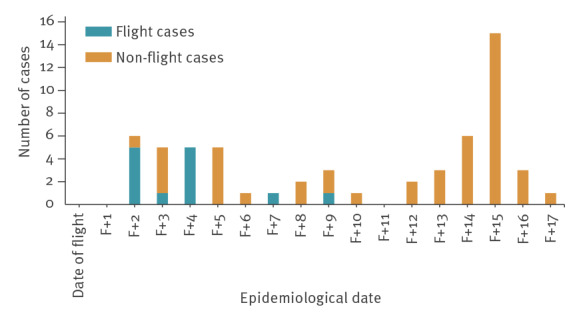
Epicurve of confirmed COVID-19 cases associated with a flight, Ireland, summer 2020 (n = 59)

Fifty-nine laboratory-confirmed cases of COVID-19 from six of the eight different health regions (Regions A–H) throughout the Republic of Ireland were linked to an international flight into Ireland in summer 2020 (Figure 1). An outbreak case was defined as positive PCR for severe acute respiratory syndrome coronavirus 2 (SARS-CoV-2) (naso-pharyngeal swab) in either a passenger or a contact of a passenger. Thirteen cases were passengers on the same flight to Ireland, each having transferred via a large international airport, flying into Europe from three different continents (Groups 1 and 2; Group 3 and Group 4). 

COVID-19: coronavirus disease.

Epidemiological date is based on the earliest available of the following dates: onset of symptoms, diagnosis, laboratory specimen collection, laboratory received date, laboratory reported date or event creation date/notification date. Onset date was available for 38, laboratory specimen collected date for 18 and diagnosis date for three cases.

Of the flight groups, Group 1 reported spending up to 12 h overnight in the transit lounge during stopover; Group 2 shared a separate transit lounge; Group 3 and Group 4 had separate short waits of under 2 h in the general airport departure area. 

**Figure 2:**
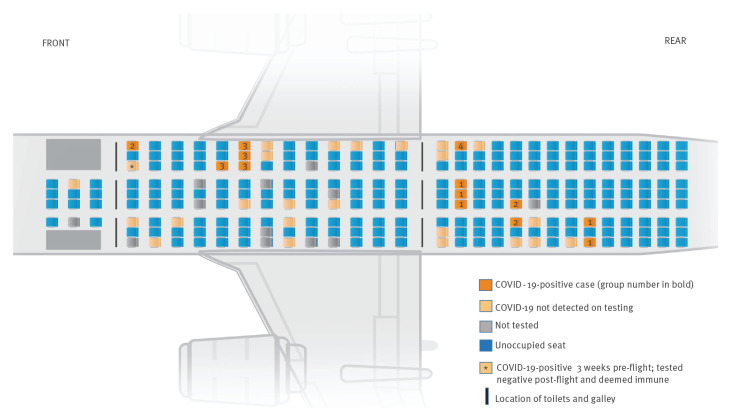
Passenger seating diagram on flight, Ireland, summer 2020 (n = 49 passengers)

The flight into Ireland was 7.5 h long and had a passenger occupancy of 17% (49/283 seats) with 12 crew (Figure 2).

COVID-19: coronavirus disease.

Numbers on the seats indicate the Flight Groups 1–4.

The age of the 13 flight cases ranged from 1 to 65 years with a median age of 23 years. Twelve of 13 flight cases and almost three quarters (34/46) of the non-flight cases were symptomatic. After the flight, the earliest onset of symptoms occurred 2 days after arrival, and the latest case in the entire outbreak occurred 17 days after the flight. Of 12 symptomatic flight cases, symptoms reported included cough (n = 7), coryza (n = 7), fever (n = 6) and sore throat (n = 5), and six reported loss of taste or smell. No symptoms were reported for one flight case. A mask was worn during the flight by nine flight cases, not worn by one (a child), and unknown for three.

**Figure 3:**
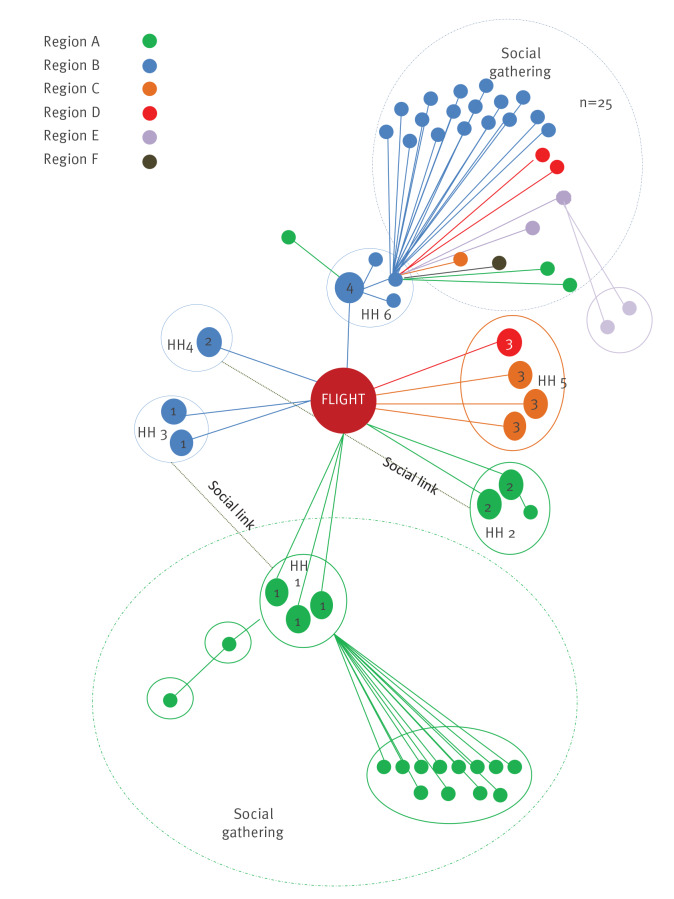
Diagram of chains of transmission, flight-related COVID-19 cases, Ireland, summer 2020 (n = 59)

COVID-19: coronavirus disease; HH: household.

The outbreak transmission pattern is described in Figure 3. Four outbreak cases were hospitalised and of these, one was admitted to the intensive care unit.

Numbers indicate the Flight Groups 1–4.

The source case is not known. The first two cases in Group 1 became symptomatic within 48 h of the flight, and COVID-19 was confirmed in three, including an asymptomatic case from this Group in Region A within 5 days of the flight. Thirteen secondary cases and one tertiary case were later linked to these cases. Two cases from Flight Group 2 were notified separately in Region A with one subsequent secondary family case, followed by three further flight cases notified from Region B in two separate family units (Flight Groups 1 and 2). These eight cases had commenced their journey from the same continent and had some social contact before the flight. The close family member of a Group 2 case seated next to the case had tested positive abroad 3 weeks before, and negative after the flight. Flight Group 3 was a household group of which three cases were notified in Region C and one case in Region D. These cases had no social or airport lounge link with Groups 1 or 2 pre-flight and were not seated within two rows of them. Their journey origin was from a different continent. A further case (Flight Group 4) had started the journey from a third continent, had no social or lounge association with other cases and was seated in the same row as passengers from Group 1. Three household contacts and a visitor of Flight Group 4 became confirmed cases. One affected contact travelled to Region E, staying in shared accommodation with 34 others; 25 of these 34 became cases (attack rate 73%) notified in regions A, B, C, D, E and F, with two cases of quaternary spread.

## Control measures

A national outbreak control team was convened with involvement of Departments of Public Health and National Focal Point to investigate, identify and interview cases, oversee contact tracing and establish control measures.

Close contact passengers were defined as two seats in every direction from the first cases notified. They were initially traced and the cabin crew were risk-assessed to establish whether they would be classified as close contacts of the cases or whether they had any illness [1,2]. 

As transmission emerged, close contacts on the flight for the first eight presenting cases were identified and nine reachable contacts tested negative. With the emergence of further positives, the remaining passengers were offered testing where contactable. Five further symptomatic positive cases were confirmed, some following medical assessment. An additional 15 passengers tested COVID-19 ‘not detected’. One passenger declined testing, and the remaining 11 passengers were not contactable. No data were available for these 11 passengers or for the crew with regard to symptomatology and subsequent illness. As a result of the risk assessment, crew members were advised to quarantine for 14 days. A ‘warn-and-inform’ letter was sent to all passengers.

The attack rate (AR) was calculated using the susceptible population of 48 passengers as a denominator, with an unknown source case among the flight cases. The maximum AR would be 12 of 48 or 25%. Reviewing the epidemiology, an AR of 17.8% was plausible, if eight of 45 contracted COVID-19 in-flight, while three cases were incubating or infected after the flight, and with one tertiary contact (date of onset F + 9 days) (Group 3). The minimum AR was 9.8%, if four of 41 passengers contracted COVID-19 in-flight (Groups 3 and 4 except one) where seven cases were incubating in-flight while one case was a tertiary contact of a flight case.

Whole genome sequencing and analysis was performed on five available samples, which came from one case travelling from one continent, three cases travelling from a different continent and one case travelling from a third continent. All five samples were identified as belonging to SARS-CoV-2 viral lineage B.1.36 (PANGOLIN nomenclature, v2.0.7). Pairwise comparison of the nucleotide sequences showed more than 99% homology across the entire viral genome, strongly suggesting a single point source of infection.

The outbreak was declared over 28 days after the last date of symptom onset.

## Discussion

This outbreak demonstrates the potential for spread of SARS-CoV-2 linked to air travel. Onward transmission from 13 passenger cases resulted in a total of 59 cases in six of eight HSE health regions in Ireland, necessitating national oversight of the outbreak. We calculated high attack rates, ranging plausibly from 9.8 % to 17.8% despite low flight occupancy and lack of passenger proximity on-board. On the flight date, 14-day COVID-19 case incidence was lower than 5 per 100,000 in Ireland, compared with 190 at time of writing (14 October 2020) [3], permitting close focus on tracing countrywide.

Exposure possibilities for flight cases include in-flight, during overnight transfer/pre-flight or unknown acquisition before the flight. The incubation period for COVID-19 may be as short as 2 days, so the potential for in-flight/airport transmission exists in this outbreak [4,5]. In-flight transmission is a plausible exposure for cases in Group 1 and Group 2 given seating arrangements and onset dates. One case could hypothetically have acquired the virus as a close household contact of a previous positive case, with confirmed case onset date less than two incubation periods before the flight, and symptom onset in the flight case was 48 h after the flight. In-flight transmission was the only common exposure for four other cases (Flight Groups 3 and 4) with date of onset within four days of the flight in all but the possible tertiary case. This case from Group 3 developed symptoms nine days after the flight and so may have acquired the infection in-flight or possibly after the flight through transmission within the household. 

The intense propagation pattern could be due to a high intensity of infection and high viral shedding in the source case as hypothesised elsewhere [6]. Case symptomatology and severity adds weight to this. Spread has been identified from asymptomatic or oligo-symptomatic cases, with similar viral loads compared to symptomatic cases [7,8], and the occurrence of pre-symptomatic transmission has been well described [9]. The extent and dynamics of asymptomatic transmission and the relative role of droplets or aerosols is unclear [10].

Rapid contact tracing can limit onward spread. This requires swift acquisition of the flight manifest, functioning contact details and enhanced surveillance of movements including transiting information. Contact details in airline manifests can be deficient [11]. In this outbreak, 11 flight passengers could not be contacted and were consequently not tested. The proposed digitalised European Union (EU) passenger locator form may assist [12].

Studies on transmission of other respiratory pathogens such as influenza A (H1N1) [13], Middle-East respiratory syndrome coronavirus [14] and SARS-CoV [15] raise the likelihood of in-flight transmission of SARS-CoV-2 [16]. In-flight symptomatic transmission has been described [17] and asymptomatic transmission hypothesised [18]. One case-series suggested an in-flight transmission rate of 3.7% [19]. Another study modelled transmission at 3.5% for train passengers seated next to a case of COVID-19 [20]. Extensive contact tracing following one symptomatic case with fever and cough, travelling on a 10 h flight in March 2020 without masks, identified a cluster of 16 cases presumed to be acquired in-flight [21]. Bohmer et al., however, note the lack of flight cases despite extensive flight contact tracing on two international flights during the incubation period of two cases [22].

Sequencing of SARS-CoV-2 can provide valuable information on virus biology, transmission and population dynamics. Genomic sequencing for cases travelling from three different continents strongly supports the epidemiological transmission hypothesis of a point source for this outbreak. The ability of genomics to resolve transmission events may increase as the virus evolves and accumulates greater diversity [23].

Non-pharmaceutical interventions [1] with distancing and restricted crew/passenger interaction can contribute to prevention of COVID-19 transmission in-flight, and recent studies speculate that face coverings confer protection [24,25]. It is interesting that four of the flight cases were not seated next to any other positive case, had no contact in the transit lounge, wore face masks in-flight and would not be deemed close contacts under current guidance from the European Centre for Disease Prevention and Control (ECDC) [1]. Following this outbreak, Ireland augmented ECDC guidance for a 3-month period to include an alert informing all passengers of a positive case on board and emphasising Ireland’s 14-day restriction-of-movement policy, in place for all those travelling from abroad, apart from a regularly reviewed shortlist of countries.

Resulting from this event, we would recommend the following: (i) When a positive COVID-19 case is linked to a flight, rapid flight contact tracing may prevent onward spread and we support the proposed EU digitalised public health passenger locator form [12] and development of improved systems of tracing. (ii) Swift action is needed where cases with no other link emerge beyond the close contact two-seat radius [1] to instigate early investigation and control measures. (iii) Enhanced surveillance should include transiting/transfer information to identify potential common links.

## Conclusion

This study is one of few thus far demonstrating in-flight transmission of SARS-CoV-2 with extensive onwards transmission. In-flight propagation patterns merit further study. Health advisories and personal responsibility, augmented by state and airline checks, should preclude travel in those with symptoms. Stringent on-board infection prevention and control measures are vital to reduce the risk of both symptomatic and asymptomatic in-flight transmission. Restriction of movement on arrival [26,27] and robust contact tracing are essential to limit propagation post-flight.
